# Trends in the Electron Microscopy Data Bank (EMDB)

**DOI:** 10.1107/S2059798317004181

**Published:** 2017-04-20

**Authors:** Ardan Patwardhan

**Affiliations:** a European Molecular Biology Laboratory, European Bioinformatics Institute, Wellcome Genome Campus, Hinxton CB10 1SD, England

**Keywords:** cryo-EM, Electron Microscopy Data Bank, EMDB, resolution, direct electron detector, electron tomography

## Abstract

Recent technological advances, such as the introduction of the direct electron detector, have transformed the field of cryo-EM and the landscape of molecular and cellular structural biology. This study analyses these trends from the vantage point of the Electron Microscopy Data Bank (EMDB), the public archive for three-dimensional EM reconstructions. Over 1000 entries were released in 2016, representing almost a quarter of the total number of entries (4431). Structures at better than 6 Å resolution now represent one of the fastest-growing categories, while the share of annually released tomography-related structures is approaching 20%. The use of direct electron detectors is growing very rapidly: they were used for 70% of the structures released in 2016, in contrast to none before 2011. Microscopes from FEI have an overwhelming lead in terms of usage, and the use of the *RELION* software package continues to grow rapidly after having attained a leading position in the field. China is rapidly emerging as a major player in the field, supplementing the US, Germany and the UK as the big four. Similarly, Tsinghua University ranks only second to the MRC Laboratory for Molecular Biology in terms of involvement in publications associated with cryo-EM structures at better than 4 Å resolution. Overall, the numbers point to a rapid democratization of the field, with more countries and institutes becoming involved.

## Abstract 2

The Electron Microscopy Data Bank (EMDB), the public archive for three-dimensional EM reconstructions, is an invaluable resource for obtaining a birds-eye view of trends affecting the field of cryo-EM. EMDB is growing rapidly, with almost a quarter of the entries having been released over the past year.

## 1. Introduction   

Recent technological advances such as the introduction of the direct electron detector have transformed the field of cryo-EM and the landscape of molecular and cellular structural biology (Kühlbrandt, 2014 ▸; Bai *et al.*, 2015 ▸; Eisenstein, 2016 ▸). Structures achieving resolutions that were once considered to be the preserve of the more established structural techniques of X-ray crystallography and nuclear magnetic resonance (NMR) are becoming a routine occurrence. At the same time, there is a greater emphasis on trying to understand the cellular context of macromolecules by placing sub-tomogram averages into tomographic reconstructions and by exploiting correlative imaging techniques (Davies *et al.*, 2011 ▸; Mattei *et al.*, 2016 ▸).

The Electron Microscopy Data Bank (EMDB; http://emdb-empiar.org) was established in 2002 (Tagari *et al.*, 2002 ▸) at the European Bioinformatics Institute (EMBL–EBI) and is the single global public repository for three-dimensional reconstructions derived from EM data (Lawson *et al.*, 2016 ▸; Patwardhan & Lawson, 2016 ▸). The EMDB contains structures determined by single-particle averaging, electron crystallo­graphy and electron tomography (ET). Its entries range from high-resolution structures in which side-chain densities are resolved to low-resolution reconstructions of cellular samples in which the distributions of biomacromolecules can be studied. The EMDB is a unique international resource and enjoys overwhelming support from the EM community. In this study, I have data-mined the EMDB and PubMed (for publications related to EMDB entries) to obtain a birds-eye perspective of the trends affecting the field. This analysis will be useful for those wanting to obtain a general idea of where the field is heading and, more concretely, for informing and justifying future investments in technology.

## 2. Methods   

This analysis is based primarily on the metadata included with the publicly released EMDB entries. The information is taken at face value and includes details about the sample, microscopy, image processing and validation (for example, the reported resolution). In order to obtain this information I used the EMDB advanced search that is available *via* a web form (http://emdb-empiar.org/emsearch) and an API, and the *EMStats* web service that provides dynamic interactive charts on the current state of the EMDB (http://emdb-empiar.org/emstats). The API queries are summarized in Table 1 ▸. Author affiliation information is not available from EMDB metadata directly. In order to obtain this information, a Python script was written to query PubMed for author affiliation information from publications related to EMDB entries. Manual cleanup of this data had to be performed in *Excel* to remove redundancy (*e.g.* ‘UK’ and ‘United Kingdom’). It should also be noted that there are limitations to the consistency of author affiliation information obtained from PubMed in terms of the format and comprehensiveness (prior to 2012 it is only available for corresponding authors, and even now it may not be provided for all authors), which may have some impact on the analysis presented. Moreover, no attempt is made to distinguish between the relative contributions of multiple authors, and all are treated equally.

## 3. Results   

**Figure 1:**
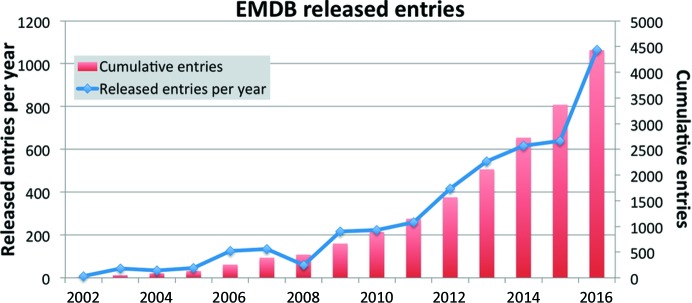
EMDB released entries. The cumulative number of released entries is shown as red bars with the *y* axis on the right-hand side. The number of entries released annually is shown as a blue marked line with the *y* axis on the left-hand side.

There were 4431 released entries by the end of 2016, of which 1065 were released in 2016: an increase of over 50% when compared with the 640 entries released in 2015, suggesting a rapid acceleration in the pace of depositions (Fig. 1 ▸). Extrapolation of the curve (*x* ^4^ curve fitted in *Excel* with *R* ^2^ = 0.9994) points to around 10 000 entries by 2020.

**Figure 2:**
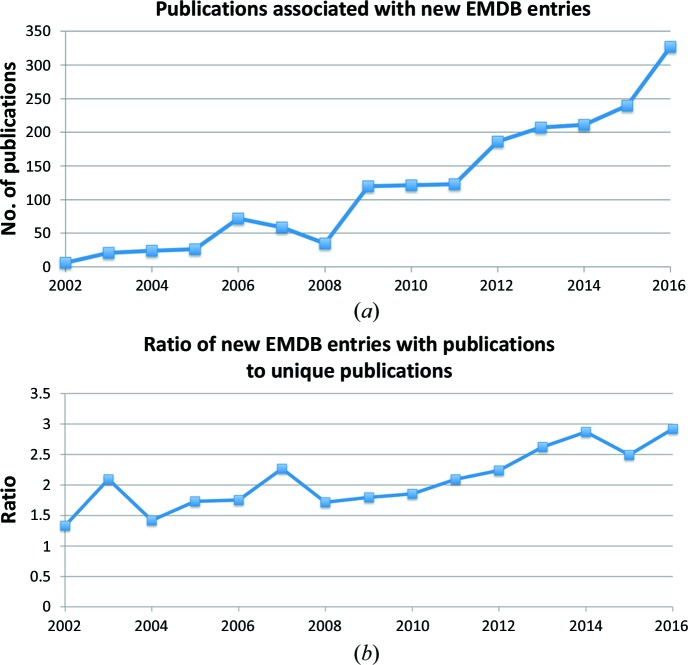
Analysis of publications associated with EMDB entries. (*a*) Count of the number of unique primary citations associated with new EMDB entries. (*b*) Ratio of the number of new EMDB entries with associated publications to the number of unique publications.

The number of publications associated with new EMDB entries increased by 25% in 2016 to over 300 (Fig. 2 ▸ *a*). The number of entries per publication continues to grow, gradually reaching ∼3 in 2017 (Fig. 2 ▸ *b*). This is indicative of the fact that a growing number of EM experiments involve the examination of related structures with small differences obtained, for instance, by using three-dimensional classification to separate the data.

**Figure 3:**
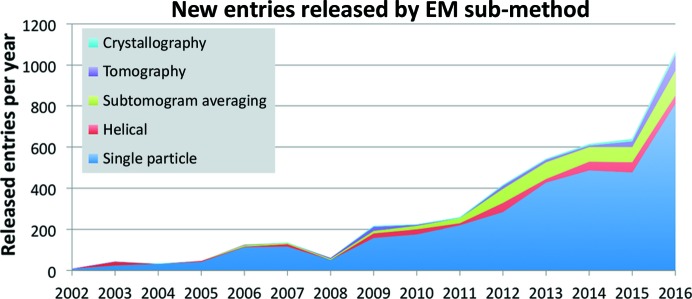
EMDB entries by EM sub-method. Stacked graph showing the number of annually released EMDB entries by sub-method category: single-particle (blue), helical (red), sub-tomogram averaging (green), tomography (purple) and crystallography (light blue).

In Fig. 3 ▸ the number of entries released per year is split between the sub-methods single-particle, helical, sub-tomogram averaging, tomography and crystallography (two-dimensional and three-dimensional). Single-particle entries continue to be the main category, with ∼76% of the total. The numbers of tomography and sub-tomogram entries have been increasing, with a 70% year-on-year increase from 2015 to 2016; the proportion of tomography-related entries as a share of the total is increasing gradually and currently stands at ∼18% of the total. The tomography category itself (*i.e.* excluding sub-tomogram averaging) has more than doubled in the past two years: eight entries in 2014, 29 in 2015 and 75 in 2016. This could indicate a greater compliance from the ET community (and enforcement by journals) to follow the recommendation derived in consensus with the community that a representative tomographic reconstruction be deposited even for studies that did not involve sub-tomogram averaging (Patwardhan *et al.*, 2012 ▸, 2014 ▸). Ribosomes and viruses continue to be the main sample categories studied, with an ∼40% share of EMDB entries (Table 2 ▸). The numbers over the past two years are in line with the long-term averages of ∼14 and 29% for ribosomes and viruses, respectively.

**Figure 4:**
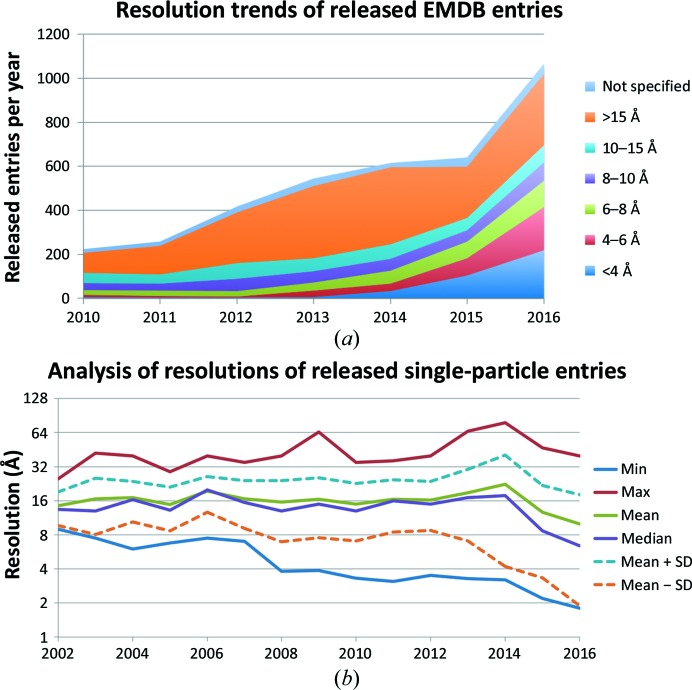
Reported resolutions of EMDB entries. (*a*) Resolution trends of released EMDB entries. Stacked graph comprised of the number of released entries separated into different resolution bands. (*b*) Statistical analysis of released EMDB entries from single-particle experiments.

Trends for different resolution bands based on the reported resolution are shown in Fig. 4 ▸(*a*). The fastest growing bands in the past two years have been the <4 Å and the 4–6 Å bands, which together comprised ∼40% of the entries in 2016. At the same time the numbers in the 8–10 and 10–15 Å bands have not experienced any appreciable growth and have decreased as a proportion of the total from an historic average of greater than 25% to ∼15%. An analysis of resolution trends for single-particle entries (Fig. 4 ▸ *b*) shows that the median resolution, which had been fairly constant until 2014 at ∼16 Å, is now on a downward trend, reaching ∼6 Å in 2016. The highest resolutions exhibit a stepwise trend, with a substantial drop in 2008 to 4 Å and then in 2015 to below 3 Å. The standard deviation of the resolution has been gradually increasing over the years, indicative of the fact that while the fraction of high-resolution structures is increasing, there are still many structures than can only be determined to low resolutions.

**Figure 5:**
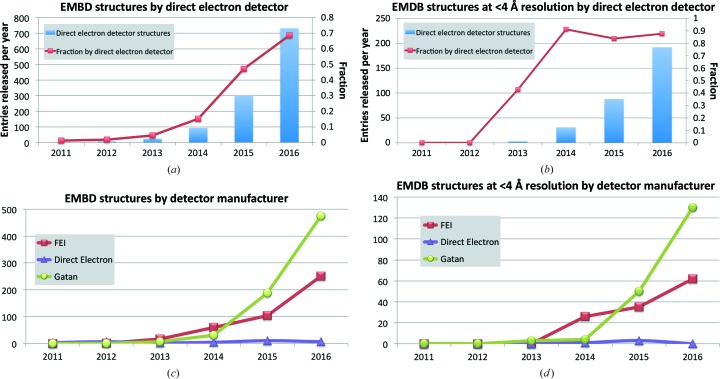
Direct electron-detector usage in EMDB entries. (*a*) Blue bars represent the number of released EMDB entries obtained using direct electron detectors (*y* axis on the left-hand side) and the red marked line represents the fraction of the total (*y* axis on the right-hand side). (*b*) The same as (*a*) but for structures at better than 4 Å resolution. (*c*) Trends for the three major direct electron-detector manufacturers: FEI, Direct Electron and Gatan. (*d*) The same as (*c*) but for structures at better than 4 Å resolution.

Fig. 5 ▸ presents trends in direct electron-detector usage. Almost 70% of the entries released in 2016 were determined using direct electron detectors and this is up from 47% in 2015 (Fig. 5 ▸ *a*). Following a rapid rise from 2012 to 2014, over 80% of the <4 Å resolution structures in the past three years were determined using direct electron detectors (Fig. 5 ▸ *b*). The Gatan K2 and the FEI Falcon II were the main cameras used (Fig. 5 ▸ *c*), and for <4 Å resolution structures over twice as many released structures in 2016 involved Gatan K2 cameras compared with the FEI Falcon II (Fig. 5 ▸ *d*).

**Figure 6:**
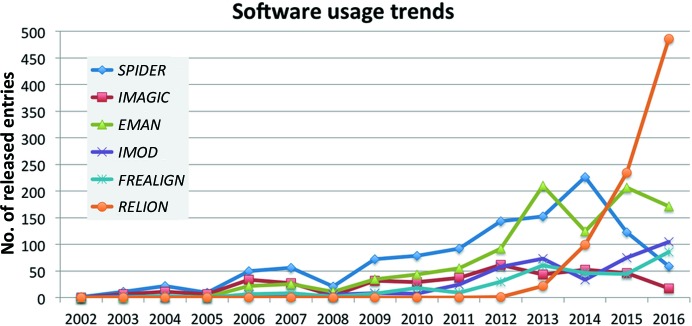
Usage trends for a selection of major EM software packages. It should be noted that the packages are not mutually exclusive and that more than one package may have been used in the same experiment.

**Figure 7:**
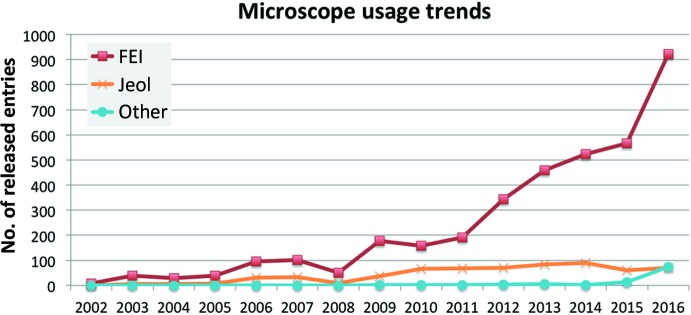
Microscope-usage trends based on microscope manufacturer. It should be noted that a substantial proportion of the ‘Other’ category are in fact FEI microscopes that have been classified incorrectly in the EMDB deposition process. There are entries where more than one microscope has been specified; however, the number of such entries is quite small and less than a handful involve the use of microscopes from different manufacturers.

Trends for a selection of major software packages used in cryo-EM are analysed in Fig. 6 ▸. The exponential rise in *RELION* (Scheres, 2012 ▸) usage clearly stands out, and it was used in almost 46% of the structures determined in 2016. The the traditional stalwarts of EM image processing, *IMAGIC* (van Heel *et al.*, 1996 ▸) and *SPIDER* (Shaikh *et al.*, 2008 ▸) appear to be stagnant or declining over the past few years in terms of usage. Even the growth in usage of *EMAN*2 (Tang *et al.*, 2007 ▸), a rising star as recently as 2013, appears to be continuing at a much more measured pace. However, it should be noted that while depositors can specify more than one software package during deposition, there is a tendency for many to only specify the main package used, leading to an underrepresentation of the use of other packages. Therefore, while it is safe to interpret the rapid rise of *RELION*, declining or stagnant trends should be treated with some caution. Two packages with smaller usage numbers but with longterm rising trends are *IMOD* (Kremer *et al.*, 1996 ▸) and *FREALIGN* (Grigorieff, 2007 ▸). The former is associated with the rising number of tomography-related entries in EMDB. Trends for microscope usage (Fig. 7 ▸) show that microscopes by FEI have an overwhelming lead that has been reinforced in recent years.

In order to study the geographic reach of cryo-EM, I have exploited author affiliation information from PubMed based on the primary citations of EMDB entries as described in §[Sec sec2]2 and the results are presented in Tables 3 ▸, 4 ▸ and 5 ▸. Table 3 ▸ shows the geographic distribution of publications associated with new EMDB entries since 2010. One clear trend is that the geographic spread has widened substantially: in 2010 there were five countries with five or more publications, whereas in 2016 there were 15. China has experienced a 13-fold increase to 43 publications in this time period, is the fourth highest producer of EM publications and has supplanted Japan as the leading Asian nation. The US has consistently been the leading nation over the analysed period, followed by Germany and the UK. The trends suggest that more recent investments in high-end EM infrastructure and people by Sweden, Australia, Singapore, Austria and the Czech Republic are starting to produce results. A similar analysis for publications associated with EMDB entries at better than 4 Å resolution is presented in Table 4 ▸. The general trends are similar: the geographic spread of the technique is widening, the US has a leading position followed by the UK, Germany and China, and high-resolution cryo-EM is growing rapidly in China. Another notable trend is that the number of publications associated with the UK is substantially greater than Germany (48 compared with 34). An institute-based analysis of publications associated with EMDB entries at better than 4 Å resolution is presented in Table 5 ▸. The two leading institutes in terms of numbers have been the MRC Laboratory of Molecular Biology (MRC–LMB) and Tsinghua University. While MRC–LMB has clearly been dominating the scene, the numbers for 2015 and 2016 are fairly similar (14 and 15 publications, respectively) and the table suggests that a substantial proportion of the growth is owing to the rapidly widening usage of high-resolution EM with the proliferation of high-end EM instrumentation and accessibility to the wider structural biology community *via* centres such as eBIC at the Diamond Light Source (Saibil *et al.*, 2015 ▸).

## 4. Discussion   

The trends in the EMDB underscore the fast-paced changes currently taking place in the cryo-EM field driven by game-changing technologies such as the direct electron detector. Headline high-resolution structures in the past few years have demonstrated the potential of the technique in a wider structural context and have prompted widespread biomedical interest and even adoption by the pharmaceutical industry [for example, the Cambridge Pharmaceutical Cryo-EM Research Consortium (including Astex Pharmaceuticals, AstraZeneca, GlaxoSmithKline, Heptares Therapeutics and UCB, MRC–LMB and the University of Cambridge’s Nanoscience Centre), Novartis, Genentech and Pfizer]. Major investments are under way to set up and expand cryo-EM facilities worldwide, which are likely to substantially increase the available capacity to produce cryo-EM structures. In fact, EMDB trends show that while the US, Germany and the UK continue to maintain leadership in the field, cryo-EM activity has risen rapidly in China to the point where they rank fourth in the number of structures being produced, and that in general a democratization has been taking place, with a substantially broadened base of countries and institutes involved (Stuart *et al.*, 2016 ▸). Furthermore, the rising number of ET structures underlines the growing importance associated with understanding biomacromolecular structure and function in the cellular context. The extrapolated value of 10 000 structures by 2020, which essentially amounts to a doubling of the number of structures being deposited yearly, therefore seems a credible prospect.

The technology trends suggest an increasing consolidation towards the use of particular solutions with a proven track record. The most common mode is the use of FEI microscopes, in particular the Titan Krios, for high-resolution cryo-EM, *RELION* for image processing and the Gatan K2 camera for high-resolution work. However, other technological solutions have also been shown to give comparable results, for example a 3.4 Å resolution alcohol oxidase structure obtained using a Jeol 3200FSC microscope (Vonck *et al.*, 2016 ▸), a 1.8 Å resolution glutamate dehydrogenase structure obtained using the *FREALIGN* software package (Merk *et al.*, 2016 ▸) and a 2.5 Å resolution *Trypanosoma cruzi* 60S ribosomal subunit structure (Liu *et al.*, 2016 ▸) obtained using a Falcon II camera. Furthermore, competitors are trying to redress the imbalance with new and improved solutions; for instance, Jeol with a new automatic specimen-loading system, a redesigned *FREALIGN* with a graphical user interface (*cisTEM*; personal communication with Timothy Grant, Janelia Research Campus), and the FEI Falcon III camera, which will even be retrofitted onto the most recently sold FEI microscope systems with a Falcon II camera. Finally, there are a number of nascent technological developments such as phase plates (Danev & Baumeister, 2016 ▸) and specimen-preparation robots [for example Spotiton (Razinkov *et al.*, 2016 ▸) from the Carragher laboratory at NYSBC and Vitrojet from the Peters laboratory at Maastricht University] that may reach a level of maturity in the next few years to significantly impact the cryo-EM field and depositions to EMDB. It may therefore be useful to repeat the analysis of EMDB trends on an annual or biannual basis to factor in such developments.

## Figures and Tables

**Table 1:** Summary of the search queries used in the analysis All queries are prefixed by http://www.ebi.ac.uk/pdbe/emdb/searchResults.html/?, *e.g.* http://www.ebi.ac.uk/pdbe/emdb/searchResults.html/?q=status:REL AND ribosom*. For the direct electron-detector queries it should be noted that for some entries more than one detector has been used and the queries may return incorrect information. All entries with multiple detectors were checked manually and the numbers were adjusted accordingly.

Query	Description	Related tables and figures
q=status:REL AND ribosom*	Search for a mention of ‘ribosom’ in all metadata fields of the released entries	Table 2 ▸
q=status:REL AND (phage OR virus)	Search for ‘phage’ or ‘virus’ in all metadata fields of the released entries	Table 2 ▸
q=status:REL AND resolution:[1 TO 4] AND NOT resolution:4	Search for released entries with a reported resolution ≥1 Å and <4 Å	Fig. 4 ▸
q=status:REL AND NOT resolution:[* TO *]	Search for released entries where the reported resolution is undefined	Fig. 4 ▸
q=status:REL AND detector:falcon	Search for released entries where an FEI Falcon camera has been used	Fig. 5 ▸
q=status:REL AND detector:("direct electron")	Search for released entries where a Direct Electron camera has been used	Fig. 5 ▸
q=status:REL AND detector:("gatan k2")	Search for released entries where a Gatan K2 camera has been used	Fig. 5 ▸

**Table 2:** Numbers and percentages of ribosome and virus-related EMDB entries

Year	Ribosomes	Viruses	All
2010	35 (16%)	69 (31%)	224
2011	47 (18%)	69 (27%)	259
2012	28 (7%)	155 (37%)	417
2013	63 (12%)	209 (38%)	544
2014	106 (17%)	125 (20%)	615
2015	101 (16%)	159 (25%)	640
2016	137 (13%)	294 (28%)	1065
Sum	517 (14%)	1080 (29%)	3764

**Table 3:** Publications associated with new EMDB entries by country Only countries with five or more publications over the period 2010–2016 are included in the table.

Country	2010	2011	2012	2013	2014	2015	2016	Sum
USA	55	50	86	116	139	135	172	753
Germany	19	25	20	22	42	53	59	240
UK	14	10	27	16	42	51	69	229
China	3	4	3	10	13	29	43	105
France	8	4	6	8	16	16	27	85
Japan	6	7	4	6	11	11	20	65
Spain	4	7	11	7	8	7	11	55
Canada	1	5	3	2	10	14	19	54
Switzerland	2	2	8	8	6	15	12	53
The Netherlands	0	1	1	3	9	15	16	45
Finland	3	1	6	6	1	5	3	25
Sweden	2	0	2	0	2	8	9	23
Australia	1	0	2	0	5	3	11	22
Singapore	0	0	3	3	2	5	6	19
Austria	0	1	2	2	4	1	7	17
Czech Republic	0	0	0	0	2	1	7	10
Israel	0	0	0	3	1	2	2	8
Russia	0	0	0	0	1	2	4	7
Belgium	0	1	0	0	3	0	2	6
Denmark	1	1	1	0	0	2	1	6
New Zealand	2	0	0	0	0	1	3	6
India	0	0	1	0	0	1	3	5
Republic of Korea	0	1	2	0	0	1	1	5
Taiwan	0	0	0	1	1	1	2	5

**Table 4:** Publications associated with EMDB entries at better than 4 Å resolution by country Only countries with three or more publications over the period 2014–2016 are included in the table.

Country	2014	2015	2016	Sum
USA	8	33	52	93
UK	8	18	22	48
Germany	6	10	18	34
China	2	8	16	26
Switzerland	1	5	4	10
France	0	5	3	8
Japan	0	1	6	7
The Netherlands	1	1	3	5
Sweden	0	2	2	4
Canada	1	0	2	3
Czech Republic	0	1	2	3
Spain	0	1	2	3

**Table 5:** Institutes of authors of publications associated with EMDB entries at better than 4 Å resolution by country Only institutes with four or more publications over the period 2014–2016 are included.

Institution	2014	2015	2016	Sum
MRC Laboratory of Molecular Biology	8	14	15	37
Tsinghua University	0	7	11	18
University of California San Francisco	0	5	6	11
University of California Los Angeles	0	7	3	10
Harvard University/Medical School	2	4	3	9
Janelia Research Campus	0	4	5	9
University of Munich	2	3	4	9
Chinese Academy of Sciences	1	3	4	8
National Cancer Institute	1	2	5	8
Max Planck Institute for Biophysical Chemistry	0	1	6	7
Purdue University	1	1	5	7
University of California Berkeley	1	1	5	7
University of Texas	3	3	1	7
University of Virginia	2	3	2	7
Columbia University	0	2	4	6
ETH	1	3	2	6
Max Planck Institute for Biochemistry	0	0	6	6
Rockefeller University	0	0	6	6
University of Washington	0	3	3	6
The Scripps Research Institute	0	1	4	5
University of Oxford	0	3	2	5
Birkbeck College	0	0	4	4
European Molecular Biology Laboratory	1	1	2	4
Lawrence Berkeley National Laboratory	1	2	1	4

## References

[bb1] Bai, X.-C., McMullan, G. & Scheres, S. H. W. (2015). *Trends Biochem. Sci.* **40**, 49–57. 10.1016/j.tibs.2014.10.005 25544475

[bb2] Danev, R. & Baumeister, W. (2016). *Elife*, **5**, e13046. 10.7554/eLife.13046 PMC4850076 26949259

[bb3] Davies, K. M., Strauss, M., Daum, B., Kief, J. H., Osiewacz, H. D., Rycovska, A., Zickermann, V. & Kühlbrandt, W. (2011). *Proc. Natl Acad. Sci. USA*, **108**, 14121–14126. 10.1073/pnas.1103621108 PMC3161574 21836051

[bb4] Eisenstein, E. (2016). *Nature Methods*, **13**, 19–22. 10.1038/nmeth.3698 27110630

[bb5] Grigorieff, N. (2007). *J. Struct. Biol.* **157**, 117–125. 10.1016/j.jsb.2006.05.004 16828314

[bb6] Heel, M. van, Harauz, G., Orlova, E. V., Schmidt, R. & Schatz, M. (1996). *J. Struct. Biol.* **116**, 17–24. 10.1006/jsbi.1996.0004 8742718

[bb7] Kremer, J. R., Mastronarde, D. N. & McIntosh, J. R. (1996). *J. Struct. Biol.* **116**, 71–76. 10.1006/jsbi.1996.0013 8742726

[bb8] Kühlbrandt, W. (2014). *Science*, **343**, 1443–1444. 10.1126/science.1251652 24675944

[bb9] Lawson, C. L., Patwardhan, A., Baker, M. L., Hryc, C., Garcia, E. S., Hudson, B. P., Lagerstedt, I., Ludtke, S. J., Pintilie, G., Sala, R., Westbrook, J. D., Berman, H. M., Kleywegt, G. J. & Chiu, W. (2016). *Nucleic Acids Res.* **44**, D396–D403. 10.1093/nar/gkv1126 PMC4702818 26578576

[bb10] Liu, Z., Gutierrez-Vargas, C., Wei, J., Grassucci, R. A., Ramesh, M., Espina, N., Sun, M., Tutuncuoglu, B., Madison-Antenucci, S., Woolford, J. L. Jr, Tong, L. & Frank, J. (2016). *Proc. Natl Acad. Sci. USA*, **113**, 12174–12179. 10.1073/pnas.1614594113 PMC5087005 27791004

[bb11] Mattei, S., Glass, B., Hagen, W. J., Kräusslich, H. G. & Briggs, J. A. (2016). *Science*, **354**, 1434–1437. 10.1126/science.aah4972 27980210

[bb12] Merk, A., Bartesaghi, A., Banerjee, S., Falconieri, V., Rao, P., Davis, M. I., Pragani, R., Boxer, M. B., Earl, L. A., Milne, J. L. & Subramaniam, S. (2016). *Cell*, **165**, 1698–1707. 10.1016/j.cell.2016.05.040 PMC4931924 27238019

[bb13] Patwardhan, A. *et al.* (2014). *Nature Struct. Mol. Biol.* **21**, 841–845. 10.1038/nsmb.2897 PMC4346196 25289590

[bb14] Patwardhan, A. *et al.* (2012). *Nature Struct. Mol. Biol.* **19**, 1203–1207. 10.1038/nsmb.2426 PMC4048199 23211764

[bb15] Patwardhan, A. & Lawson, C. L. (2016). *Methods Enzymol.* **579**, 393–412. 10.1016/bs.mie.2016.04.015 PMC5351295 27572735

[bb16] Razinkov, I., Dandey, V. P., Wei, H., Zhang, Z., Melnekoff, D., Rice, W. J., Wigge, C., Potter, C. S. & Carragher, B. (2016). *J. Struct. Biol.* **195**, 190–198. 10.1016/j.jsb.2016.06.001 PMC5464370 27288865

[bb17] Saibil, H. R., Grünewald, K. & Stuart, D. I. (2015). *Acta Cryst.* D**71**, 127–135. 10.1107/S1399004714025280 PMC4304693 25615867

[bb18] Scheres, S. H. W. (2012). *J. Struct. Biol.* **180**, 519–530. 10.1016/j.jsb.2012.09.006 PMC3690530 23000701

[bb19] Shaikh, T. R., Gao, H., Baxter, W. T., Asturias, F. J., Boisset, N., Leith, A. & Frank, J. (2008). *Nature Protoc.* **3**, 1941–1974. 10.1038/nprot.2008.156 PMC2737740 19180078

[bb20] Stuart, D. I., Subramaniam, S. & Abrescia, N. G. (2016). *Nature Methods*, **13**, 607–608. 10.1038/nmeth.3946 PMC10720754 27467721

[bb21] Tagari, M., Newman, R., Chagoyen, M., Carazo, J. M. & Henrick, K. (2002). *Trends Biochem. Sci.* **27**, 589. 10.1016/s0968-0004(02)02176-x 12417136

[bb22] Tang, G., Peng, L., Baldwin, P. R., Mann, D. S., Jiang, W., Rees, I. & Ludtke, S. J. (2007). *J. Struct. Biol.* **157**, 38–46. 10.1016/j.jsb.2006.05.009 16859925

[bb23] Vonck, J., Parcej, D. N. & Mills, D. J. (2016). *PLoS One*, **11**, e0159476. 10.1371/journal.pone.0159476 PMC4961394 27458710

